# Epidermal cells and sensory neurons team up

**DOI:** 10.7554/eLife.107113

**Published:** 2025-05-12

**Authors:** Jean-Christophe Boivin, Tomoko Ohyama

**Affiliations:** 1 Department of Biology, McGill University Montreal Canada

**Keywords:** mechanosensation, epidermis, nociception, somatosensation, *D. melanogaster*

## Abstract

Experiments in fruit fly larvae show that epidermal cells can communicate with sensory neurons to drive responses to pain.

**Related research article** Yoshino J, Mali SS, Williams CR, Morita T, Emerson CE, Arp CJ, Miller SE, Yin C, Thé L, Hemmi C, Motoyoshi M, Ishii K, Emoto K, Bautista DM, Parrish JZ. 2025. *Drosophila* epidermal cells are intrinsically mechanosensitive and modulate nociceptive behavioral outputs. *eLife* **13**:RP95379. doi: 10.7554/eLife.95379.

The outer layer of the skin, known as the epidermis, is a structure found throughout the animal kingdom. As well as acting as a protective barrier against harmful environmental stimuli, the epidermis is increasingly thought to be important for sensing touch and pain. Indeed, recent studies in vertebrates have shown that, as well as responding directly to physical contact, the epidermis can modulate the activity of sensory neurons involved in perceiving touch and pain (Woo et al., 2014; Shindo et al., 2021; Mikesell et al., 2024; Xu et al., 2022).

A similar phenomenon has been observed in fruit fly larvae, which have a remarkably simple sensory system that is conserved across species (Liu and Zhang, 2022; He et al., 2022; Hehlert et al., 2021). In fruit flies, noxious mechanical stimuli trigger the larvae to display escape behaviors such as bending into a ‘C’ shape and rolling away (Hwang et al., 2007). Previous studies revealed that a protective layer of epidermal cells surrounding the ends of sensory neurons can alter how larvae respond to painful mechanical inputs, with more ‘ensheathed’ neurons showing greater sensitivity to pain (Luedke et al., 2024; Mauthner et al., 2021). However, it remained unclear whether mechanical cues directly activate epidermal cells and how this activation affects sensory neurons. Now, in eLife, Kazuo Emoto, Diana Bautista, Jay Parrish and colleagues – including Jiro Yoshino, Sonali Mali, and Claire Williams as joint first authors – report new findings that help to answer these questions (Yoshino et al., 2025).

To test whether epidermal cells directly influence sensory neurons, the team (who are based at various institutes in the United States and Japan) used light and heat-activated genetic tools to stimulate these cells. This activation triggered a wide array of escape behaviors typically associated with touch and pain, which could be reduced by silencing specific sensory neurons. Monitoring of calcium levels showed that stimulation of epidermal cells increased the ongoing activity of pain-sensing neurons. Notably, using lower intensity tools to activate epidermal cells made the larvae more responsive to future mechanical stimulation, through a process known as sensitization. This meant that mechanical stimuli that would not normally trigger a strong behavioral response in the larvae were now able to trigger one. Taken together, these findings indicate that epidermal cells can directly interact with sensory neurons to drive behavioral responses to mechanical stimuli and make the neurons more sensitive to subsequent mechanical stimuli.

Recent studies have shown that epidermal cells can be activated by the mechanical stress induced by stretching (Shindo et al., 2021; Mikesell et al., 2022). Building on this, Yoshino et al. confirmed that the epidermal cells in fruit fly larvae are also stretch-sensitive, as evidenced by them experiencing an increase in calcium activity. To identify how this leads to escape behaviors, the team studied proteins in the epidermal cells that might transform mechanical signals into activity. They found that a calcium channel known as Orai, which allows calcium to enter cells, is a critical player in this process. The rise in calcium caused by Orai prompts epidermal cells to release vesicles containing molecules that modulate neuronal activity (Figure 1A-C).

**Figure 1.**
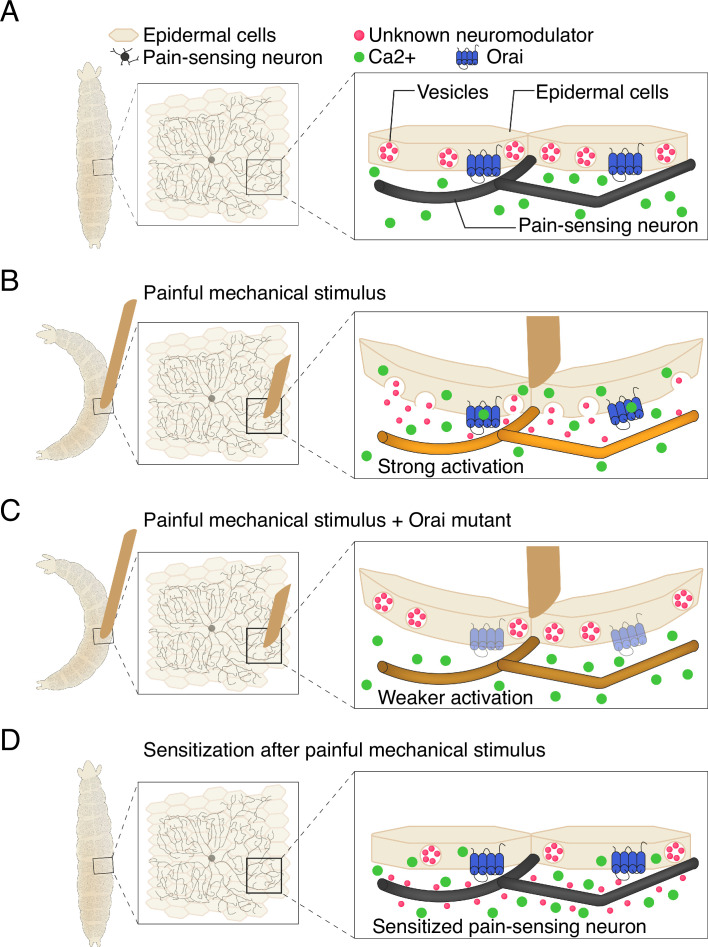
Communication between epidermal cells and pain-sensing neurons in fruit fly larvae. (**A**) Fruit fly larvae (shape on left hand side) are covered in pain-sensing neurons (black) that lie ensheathed beneath epidermal cells (beige). Within each epidermal cell are vesicles containing molecules that influence neuron behavior (neuromodulators; pink circles), as well as protein channels called Orai (blue) that transmit calcium ions (green), which sit inactive at the cell surface (**B**) A painful mechanical stimulus (depicted as brown rectangular shape) elicits a response that causes the larvae to bend into a C-shape. The stimulus causes calcium to flow into epidermal cells via the Orai channels and trigger the release of neuromodulators (the specific nature of which remains unknown) from vesicles. The combination of intrinsic neuronal properties and the presence of neuromodulators strongly activates pain-sensing neurons. (**C**) When Orai is mutated to be non-functional, epidermal cells cannot take in calcium, which prevents the release of neuromodulators, weakening the activation of the sensory neurons. (**D**) Once a painful mechanical input has been applied, the neuromodulators released by epidermal cells ensure that pain-sensing neurons remain sensitive to the stimuli – a process known as sensitization. If a larva receives subsequent painful cues, the neurons will be more easily activated and ready to facilitate escape behaviors.

These findings led Yoshino et al. to propose a model in which epidermal cells serve as key components of the first steps of sensory processing. Mechanical stress raises intracellular calcium levels in epidermal cells via the Orai channel, triggering the release of vesicles (Figure 1B). The content of these vesicles then modifies the function of sensory neurons, causing them to drive the escape behaviors displayed by the larvae. Importantly, the model also suggests that activation of epidermal cells leads to increased neuronal and behavioral responses to subsequent mechanical stimuli (Figure 1D). Notably, Yoshino et al. found that the epidermal cells do not need to ensheath sensory neurons in order to drive escape behaviors, although this configuration likely facilitates communication.

Collectively, this study sheds new light on how epidermal cells transform mechanical stimulation into activity in pain-sensing neurons. While the specific molecules released from the mechanically stressed epidermal cells are yet to be identified, Yoshino et al. propose that they could be inflammatory molecules or neuromodulators. Future research building on this work will likely clarify these mechanisms, leading to a more comprehensive understanding of sensory processing.
